# F. B. Vandegrift & Co.’s Handbook of the United States Tariff

**Published:** 1894-09

**Authors:** 


F. B. VANDEGRIFT & CO.'S HANDBOOK OF THE UNITED STATES TARIFF, containing the Customs Tariff Act of 1894, with complete schedules. F. B. Vandegrift & Co., 27 William Street, New York, and 50 South Fourth Street, Philadelphia. Price, $1.50.

The advent of this book was announced in this journal in the July number, page 224. It contains over 12,000 articles giving rate of duty, paragraph of the law and decisions of the courts, General Appraisers, and Treasury Department. It also contains complete list of articles on which drawback has been allowed with the amount of wastage. Copies can be had of the publishers, F. B. Vandegrift & Co.

